# Assessing Audience Members' Ability to Identify the Media Source of a Health Campaign Disseminated via Different Media

**DOI:** 10.3389/fpubh.2018.00196

**Published:** 2018-07-19

**Authors:** Simone Pettigrew, Michelle Jongenelis, Fiona Phillips, Terry Slevin, Vanessa Allom, Stacey Keightley, Sarah Beasley

**Affiliations:** ^1^School of Psychology, Curtin University, Perth, WA, Australia; ^2^Cancer Council Western Australia, Subiaco, WA, Australia

**Keywords:** campaign evaluation, mass media, campaign recall, television, digital media

## Abstract

**Background:** An important criterion for health campaign media selection is the ability to achieve campaign awareness among target audiences. However, existing campaign exposure metrics cannot be applied across both traditional and digital media, which complicates decision making. The present study assessed the validity of using self-report as a measure of the extent to which different types of media achieve campaign awareness to assist in determining appropriate media budget allocations.

**Methods:** A quasi-experiment involving varying combinations of television, online video, and online display smoking cessation advertisements was conducted to determine whether audience members were able to accurately report the source of their exposure to the campaign.

**Results:** Of the 719 Western Australian adults sampled (50% males, 50 females, 50% smokers, 50% non-smokers), 64% reported seeing the campaign in the previous 2 weeks. Of these, 91% reported seeing the advertisement on television, 8% on online video, and 21% on online display (respondents could select multiple media). Despite proportional scheduling of the three media over the discrete campaign periods, in most cases respondents assumed their exposure had occurred via television, regardless of the actual source of exposure.

**Conclusions:** Among both smokers and non-smokers, television had primacy in memory regardless of the actual media used. As such, relying on self-reported recall is unlikely to constitute a reliable method of assessing target audience exposure to campaigns on different media where those media are all screen-based. The results highlight the need for alternative media effectiveness metrics that permit direct comparisons between traditional and digital media.

## Introduction

Strategic media selection is crucial for optimizing campaign dissemination to target audiences, especially in the context of highly constrained promotion budgets. Historically, campaign managers have selected from a limited range of media, most commonly television, radio and print. Of these, television has been considered most effective in reaching audiences (1), with correlations demonstrated between television advertising expenditure and calls to quit help lines (2, 3). However, in recent years the media environment has become increasingly fragmented, and numerous forms of digital media (e.g., websites, social media, YouTube) are commonly used for message dissemination (4).

In Australia, five analog television stations have been replaced by more than 20 broad-coverage digital stations and numerous other digital stations operating in specific geographical regions. Many of these can be accessed via the internet on a range of electronic devices, including in “catch-up” mode that allows viewing at any time. In addition, there are purely online channels such as YouTube that provide video content that is preceded by advertisements. Given this large-scale fragmentation of media formats, there is now substantial uncertainty about how limited advertising budgets should be allocated to different media (5).

Addressing this issue is difficult because methods traditionally used to assess message exposure and awareness (e.g., target audience rating points, reach, frequency) do not suit digital media, which precludes direct comparisons and complicates media selection decisions (4). One method of overcoming this problem is to ask target audience members to self-report if and where they were exposed to campaign messages. This approach relies on people's ability to recall the message and where they saw/heard it. While this approach may have been more practical when media were substantively different (e.g., moving pictures on television vs. audio on radio and static images/text for press), the evolution of digital media means that multiple media are screen-based, which may make it increasingly difficult for audiences to differentiate the message source (6). However, little is known about how audiences process different forms of media and the implications for their recall of how they were exposed to campaign messages. To address this research gap and assist in determining optimal media budget allocations, the aim of the present study was to test the extent to which audiences are able to identify the medium of exposure to a smoking cessation campaign.

## Methods

The study was part of a larger tobacco campaign research project (7), and involved assessing the validity of self-report as a measure of campaign exposure among various forms of screen-based media. The media selected for inclusion in the study were: television (TV), online video (OV: e.g., pre-rolls shown before catch-up television episodes and video clips played on YouTube and news websites), and online display (OD: e.g., banner ads). The choice of television and online media reflects the prevalence of their use by audiences; around 85% of Australians watch commercial television (8) and use the Internet (9).

The advertisements used in the study were from the Western Australian “16 Cancers” campaign that focused on (i) the links between smoking and numerous cancers and (ii) the ability of smokers to reduce their risk of developing these cancers by quitting smoking. Emotionally intense images depicted people experiencing the effects of five specific types of cancers (lung, bowel, bladder, throat and stomach cancers), while a voiceover explained that 16 difference cancers have been associated with smoking (further details of the campaign available at https://makesmokinghistory.org.au/more-information/past-campaigns/16-cancers). Two 30-s variations of the ads included three of the five scenes and three 15-s ads each featured one scene. These advertisements were used for the television and online video conditions, while the online display condition featured static images from the advertisements and related graphics that appeared on a range of websites and social media.

A quasi-experimental design was adopted that involved broadcasting advertisements using all possible campaign media combinations involving TV, OV, and/or OD formats. This resulted in the following seven conditions: TV only, OV only, OD only, TV + OV, TV + OD, OV + OD, and TV + OV + OD. During the 13-week campaign period, advertisements were aired via randomly selected combinations of these conditions for 1 week at a time.

The advertising agency was instructed to arrange scheduling of each condition to reflect approximately equal levels of expected reach, based on best estimates given the lack of equivalent reach measures across the different media. This resulted in the patterns of expenditure (including both production and broadcasting costs) and resulting campaign awareness levels shown in Table 1. Reflecting the higher costs of developing video content, the TV and OV conditions had substantially higher costs in total and per percentage point of campaign awareness than the OD condition.

**Table 1:** Media costs by condition.

**Media Condition**	Costs^*^ ($AUD)			**Awareness %**	**Cost per % point of awareness ($AUD)**
	**Production**	**Broadcasting**	**Total**		
TV	184,493	68,496	252,989	66	3833
TV+OV	188,970	110,426	299,397	77	4536
OD	1,932	26,361	28,293	41	429
OV+OD	187,622	68,291	255,914	67	3877
TV+OD	186,425	94,857	281,282	60	4262
TV+OV+OD	190,902	136,787	327,689	73	4965
OV	185,690	41,930	227,621	1	3449

* *The cost derivation process is explained in detail in Allom et al. (7), from which this table is adapted*.

The *16 Cancers* campaign had run across all three media in the most recent campaign wave (completed 3 weeks earlier) to minimize confounding due to the order of media conditions and a lack of prior familiarity with the campaign. Based on Quitline call data showing that the level of call activity reverts to baseline around 1 week after campaign advertising ceases (10) and research indicating that stimuli recall (including advertising stimuli) can decrease substantially over the week following exposure (11, 12), 1-week breaks were scheduled between each media block to allow time for the campaign effects to dissipate. A telephone survey was administered by an accredited social research agency to assess awareness of the campaign among Western Australians aged 25-54 years (*n* = 719). Using random digit dialing of landlines, ~100 respondents were contacted during each of the seven “off” weeks and administered the same questionnaire. Quotas were used to recruit a sample comprising 50% males (50% females) and 50% smokers (50% non-smokers). Smokers constituted the campaign′s primary audience because of the focus on encouraging cessation, and non-smokers were a secondary audience due to the need to encourage non-smokers to promote quit attempts among friends and family who smoke and to reinforce the decision not to smoke among quitters (13).

Respondents were asked if they could remember seeing any quit smoking advertisements during the previous 2 weeks. This time period was important to ensure they were cued to only consider ads they had seen within the time period of the relevant experimental condition. To assess recall, those who answered in the affirmative were asked to describe the advertisement (to confirm they had seen the *16 Cancers* campaign) and nominate the media on which they had seen it. To assess recognition, those who did not demonstrate recall had the campaign described to them and asked if they had seen it in the previous 2 weeks. If answering in the affirmative, they were asked to nominate all the relevant media on which they had seen it. Awareness was calculated by summing recall and recognition. The study protocol received ethics clearance from a University Human Research Ethics Committee. Further information relating to ethics approval, study design, and the survey items is located in the supplementary materials.

## Analysis

The likelihood of respondents stating the correct media type was assessed by comparing the proportion of respondents reporting seeing the ad on a particular medium in the weeks when that medium was actually used to the proportion reporting they saw it on that medium when it was not used. Specifically, a series of 2 × 2 Pearson chi-square analyses was conducted to determine whether awareness of each media type (TV, OV, OD) differed in the weeks it was used compared to the weeks it was not (i.e., TV used vs. TV recalled; OV used vs. OV recalled; OD used vs. OD recalled). The frequency of nominating each type of media was also assessed according to the number of different media used in any one campaign period to determine whether increasing the number of media resulted in greater confusion. Wilcoxon signed rank tests were used to assess differences in awareness between the nominated media (i.e., TV awareness vs. OV awareness; TV awareness vs. OD awareness; OV awareness vs. OD awareness).

## Results

In total, 64% of respondents reported awareness of the *16 Cancers* campaign over the two weeks prior to completing the survey (ranging from 41% for the OD condition to 77% for the TV+OV condition). As shown in Table 2, regardless of the media used within the previous 2 weeks, respondents who were aware of the campaign were most likely to nominate television as a medium via which they were exposed. Across the campaign periods when the television advertisements were aired, 91% of respondents demonstrating awareness of the campaign nominated this medium. This proportion did not change significantly among those reporting exposure to the campaign in the periods when television was not used (90%, *p* = 0.870).

**Table 2:** Media used vs. respondents′ reported media exposure.

		**Media type recalled by respondents**		
	**Campaign awareness %**	**Television (TV) *n* (%)**	**Online Video (OV) *n* (%)**	**Online Display (OD) *n* (%)**
**TYPE OF MEDIA USED IN CAMPAIGN (SAMPLE SIZE)**				
TV (weeks 1, 3, 9, 11: *n* = 405)	68	249 (91)^a^^b^	18 (7)^b^	62 (23)
Non-TV (weeks 5, 7, 13: *n* = 314)	60	170 (90)^a^^b^	17 (9)^c^	35 (19)
OV (weeks 3, 7, 11, 13: *n* = 416)	69	254 (89)^a^^b^	20 (7)^b^	64 (23)
Non-OV (weeks 1, 5, 9: *n* = 303)	58	165 (93)^a^^b^	15 (9)^c^	33 (19)
OD (weeks 5, 7, 9, 11: *n* = 413)	63	232 (89)^a^^b^	23 (9)^b^	55 (21)
Non-OD (weeks 1, 3, 13: *n* = 306)	66	187 (93)^a^^b^	12 (6)^b^	42 (21)
All campaign weeks combined (*n* = 719)	64	419 (91)^a^^b^	35 (8)^b^	97 (21)
**NUMBER OF MEDIA USED (SAMPLE SIZE)**				
**1 media condition**				
TV only (*n* = 101)	60	59 (97)^a^^b^	5 (8)	11 (18)
OV only (*n* = 102)	60	59 (97)^a^^b^	2 (3)^c^	11 (18)
OD only (*n* = 102)	52	48 (91)^a^^b^	7 (13)	7 (13)
**2 media condition**				
TV + OV (*n* = 103)	78	69 (86)^a^^b^	5 (6)^b^	20 (25)
TV + OD (*n* = 100)	63	58 (92)^a^^b^	3 (5)^c^	15 (24)
OV + OD (*n* = 110)	67	63 (85)^a^^b^	8 (11)	17 (23)
**3 media condition**				
TV + OV + OD (*n* = 101)	69	63 (90)^a^^b^	5 (7)^c^	16 (23)

a *Significantly different from OV at p < 0.001*.

b *Significantly different from OD at p < 0.001*.

c *Significantly different from OD at p < 0.01*.

By comparison, few respondents reported seeing the ads via online video or online display in either the weeks when these media were used (OV = 7%, OD = 21%) or when they were not (OV = 9%, OD = 21%). Despite these low levels of awareness for OV and OD, overall campaign awareness was comparable across campaign periods (Table 2, column 2), indicating that exposure was consistent across all media but television was usually remembered as the medium of exposure, whether this was correct or not. This pattern of results was evident regardless of the number of media in use, suggesting this factor had minimal effect on respondents′ ability to nominate a correct medium of exposure.

## Discussion

The aim of this study was to assess whether viewers were able to accurately remember the media via which they were exposed to a smoking cessation campaign. This information is useful given the importance of campaign evaluation (14), the high relative costs of television compared to digital media (15), and the existence of incompatible metrics for determining the relative effectiveness of different media in reaching target audiences (4). The results have relevance for campaign developers and administrators in their efforts to determine the most effective approaches to media selection and campaign evaluation.

The results suggest that television had primacy in memory regardless of the actual media used. As such, relying on self-report is likely to constitute an unreliable method of assessing audience exposure to smoking cessation campaigns on different media where those media are all screen-based. When examining the media awareness results in aggregate across the total campaign period, the overall trends would lead campaign managers to assume that television is vastly superior to digital media in achieving awareness. However, by manipulating the advertising scheduling to allow analyses by media type, it was apparent that this may not always be the case. This outcome is relevant to campaign administrators who need to ensure that the relative reach of different types of media is fully appreciated in media selection and scheduling decisions.

A strength of the present study was the real-world application of a campaign awareness experiment to provide insight into how audiences process media-related information. The results highlight the importance of developing alternative media reach metrics that facilitate direct performance comparisons between traditional and digital media. In the meantime, it remains necessary to continue using measures such as target audience rating points for television and other metrics such as website visits and click-through rates for digital media.

Several notable limitations should be considered when interpreting these findings. A major potential confounder is the nature of the experimental design that involved administering the different media conditions to the same population. Although efforts were made to minimize the effects of prior exposure by limiting the recall period to 2 weeks, it may have been difficult for respondents to isolate their recall to that specific timeframe. It was not possible to use other states as controls because of different smoking cessation campaigns running in these locations. Other limitations include the small range of media included, the use of only one type of smoking cessation message, and the lack of data relating to audience members′ media usage patterns. To build on the results of this study, future research could seek to overcome the experimental limitations by conducting a controlled trial across geographical regions that permits exposure of different audiences to a single media condition to provide a better indication of the relative effects of each condition.

## Author contributions

SP took primary responsibility for preparing the manuscript. MJ performed the analyses. TS, SP, MJ, VA, and SK conceptualized the study. FP and SB managed data collection; all authors assisted with manuscript preparation and approved the final manuscript for submission.

### Conflict of interest statement

The authors declare that the research was conducted in the absence of any commercial or financial relationships that could be construed as a potential conflict of interest.
