# Supplementary material for: Assessing Audience Members' Ability to Identify the Media Source of a Health Campaign Disseminated via Different Media
Source: Front Public Health. 2018 Jul 19;6:196. doi: 10.3389/fpubh.2018.00196 (PMC6060251; doi:10.3389/fpubh.2018.00196)
Supplement: Supplementary file 1 [file Image_1.PDF]

Assessing audience members' ability to identify the media source of a health campaign disseminated via different media

Supplementary Materials

Ethics information

Ethics clearance was obtained from the Curtin University Human Research Ethics Committee. Respondents to the telephone survey provided verbal consent to participating in the study and were assured that "All the information you provide will remain confidential".

Study design

Figure 1 shows the media allocations across the study period.

Figure 1: Media schedule

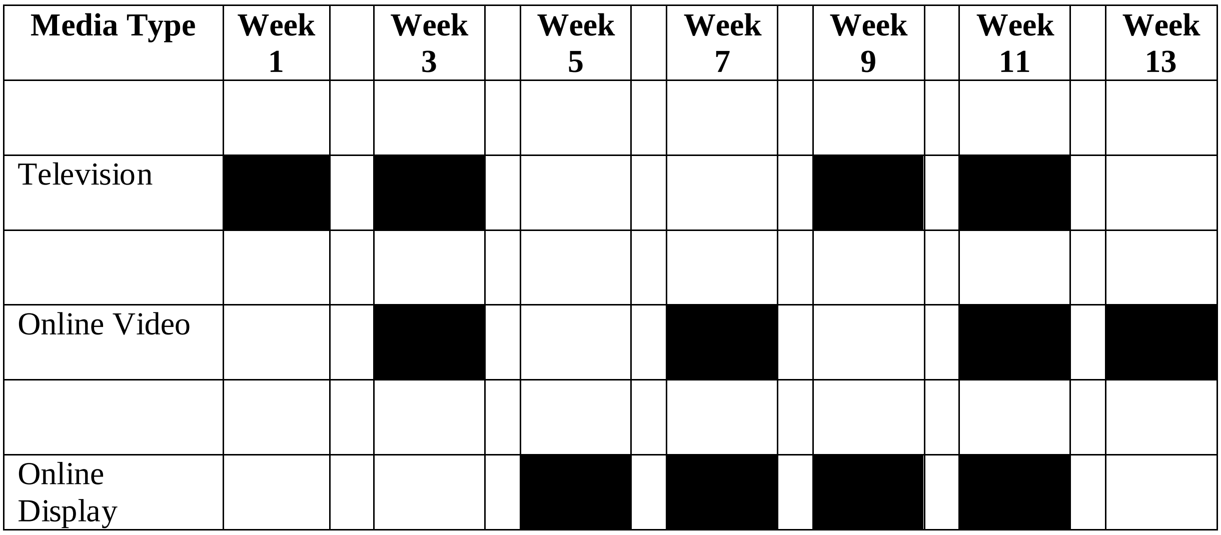

Smoking status and campaign recall and recognition questionnaire items:

"Do you smoke cigarettes regularly, that is, at least one cigarette a day or seven per week?"

"During the last two weeks – that is since XXXX, have you seen any advertising about smoking?"

If 'yes':

- "Please tell me everything you can remember about the ad."

- "Could you please list the places you have seen the ad, starting with where you have seen it the most? – Anywhere else?

  If TV, OV, or OD not mentioned:

  - "Do you remember seeing this advertisement on television in the last two weeks, that is since XXXX?"

  - "Do you remember seeing the advertisement playing on a website before or during a video online, in the last two weeks, that is since XXXX?"

  - "Do you remember seeing an advertisement on Facebook in the last two weeks, that is since XXXX, which shows people experiencing the effects of smoking-related cancers?"

If 'no':

- "Do you remember seeing a television advertisement about smoking in the last two weeks, that is since XXXX, which includes a series of scenes with people experiencing the effects of smoking-related cancers? For example, a man being diagnosed with lung cancer, a man having surgery for bowel cancer and a woman eating via a tube in the stomach due to stomach cancer."

  If 'yes':

  - "Could you please list the places you have seen the ad, starting with where you have seen it the most? – Anywhere else?

  If TV, OV, or OD not mentioned:

    - "Do you remember seeing this advertisement on television in the last two weeks, that is since XXXX?"

    - "Do you remember seeing the advertisement playing on a website before or during a video online, in the last two weeks, that is since XXXX?"

    - "Do you remember seeing an advertisement on Facebook in the last two weeks, that is since XXXX, which shows people experiencing the effects of smoking-related cancers?"
